# Understanding Statin Non-Adherence: Knowing Which Perceptions and Experiences Matter to Different Patients

**DOI:** 10.1371/journal.pone.0146272

**Published:** 2016-01-25

**Authors:** Hans Wouters, Liset Van Dijk, Harm C. J. Geers, Nina A. Winters, Erica C. G. Van Geffen, Anne M. Stiggelbout, Marcel L. Bouvy

**Affiliations:** 1 Department of Pharmacy, Unit of Pharmacotherapy and Pharmaceutical Care, Groningen Research Institute of Pharmacy, Faculty of Mathematics and Natural Sciences, University of Groningen, Groningen, The Netherlands; 2 NIVEL, Netherlands Institute for Health Services Research, Utrecht, The Netherlands; 3 Division of Pharmaco-epidemiology and Clinical Pharmacology, Utrecht Institute for Pharmaceutical Sciences, Faculty of Science, Utrecht University, Utrecht, The Netherlands; 4 Department of Medical Decision Making, Leiden University Medical Center, Leiden, The Netherlands; University of Milan, ITALY

## Abstract

**Background:**

Non-adherence to statins is substantial and is associated with numerous perceptions and experiences. However, time limits in clinical practice constrain in depth explorations of these perceptions and experiences.

**Objectives:**

To propose and examine a strategy aimed at an efficient assessment of a wide array of perceptions and experiences regarding the efficacy, side effects, and practical problems of statins. Furthermore, to assess associations between this wide array of experiences and perceptions and non-adherence and to examine whether patients' 'perceived self-efficacy' moderated these associations.

**Methods:**

Patients were recruited through community pharmacies. A wide array of specific patient perceptions and experiences was efficiently assessed using the electronic Tailored Medicine Inventory that allows people to skip irrelevant questions. Adherence was measured through self-report and pharmacy refill data.

**Results:**

Of the two-hundred twenty-nine patients who participated (mean age 63.9, standard deviation 10.2), 40%-70% doubted the necessity of or lacked knowledge about the efficacy of statins, 20%-35% of the patients were worried about joint and muscle side effects or had experienced these, and 23% had encountered practical problems regarding information about statins, intake of tablets, the package, or the blister. Experiencing more practical problems was associated with increased *un*intentional non-adherence (Odds ratio 1.54, 95%CI:1.13–2.10, P < 0.01), whereas worrying about side effects was associated with increased *in*tentional non-adherence (Odds ratio 1.90, 95%CI:1.17–3.08, P < 0.01). Higher 'perceived self-efficacy' did not moderate these associations.

**Conclusions:**

Insight into patients' specific barriers with regard to appropriate statin use may reveal personal reasons for being non-adherent. The Tailored Medicine Inventory is a promising tool to devise individualized intervention strategies aimed at improving adherence by the clinician-patient alliance.

## Introduction

Statins are an effective therapy for the primary and secondary prevention of cardiovascular disease. [1] At the same time, physicians and pharmacists are faced with the challenge of patient non-adherence to statin therapy. Not only is non-adherence substantial, as it ranges from 30%-60% at 6 to 12 months after therapy initiation [1], its causal structure is also likely to be multifactorial with different predictors being important for different patients. [2] Adherence improving interventions should therefore be tailored to individual patients' needs and circumstances and require comprehensive insight into various potential barriers to patients' appropriate use of statins. However, time and other constraints in clinical practice pose obstacles for physicians and pharmacists to understand individual patient barriers.

In this study, we therefore propose and examine a strategy to assess a wide array of patient specific perceptions and experiences in a manner that is both comprehensive and efficient. Specifically, we adopted the Tailored Medicine Inventory (TMI) that was previously used to assess perceptions and experiences with regard to the efficacy, side effects, and practical problems encountered by patients who were treated with antidepressants [3] and women who were treated with endocrine therapy for prevention of breast cancer recurrence. [4] By adopting logical routes, the TMI enables skipping of side effects and practical problems that are not applicable to an individual patient. This enables researchers to include many different potential predictors of non-adherence while test burden can also be kept at a minimum for patients who encounter only few problems.

Findings of the TMI about specific perceptions and experiences with regard to statin use are likely to be complementary to findings from other studies which have adopted a patient-oriented perspective, but which have focused on more general patient beliefs about statins. [1,5]

Specifically, we examined whether one of such more distal patient beliefs, namely 'perceived self-efficacy', compensated for the impact of side effects and practical problems and as such weakened the association of specific adverse perceptions and experiences with non-adherence. A patient's 'perceived self-efficacy' can be defined as his or her perceived ability or confidence to execute a particular behavior. In our study, we focused on 'perceived self-efficacy' with regard to 'learning about' and 'taking of' statins. Higher levels of 'perceived self-efficacy' with regard to learning about and taking of statins were previously found to be associated with better adherence. [6] We therefore hypothesized higher levels of 'perceived self-efficacy' to at least partially undo the negative impact of adverse experiences on adherence. Finally, we also distinguished between 'unintentional non-adherence' due to forgetting, and 'intentional non-adherence' or more conscious unwillingness to take statins.

Accordingly, the aims of this study were to comprehensively and efficiently examine patients' perceptions and experiences, their 'perceived self-efficacy', as well as associations with non-adherence.

## Material and Methods

### Participants

For this cross-sectional study, patients were recruited through community pharmacies between January and August 2012 and were included if they were being, or had been treated with statin therapy in the year prior to recruitment. For ethical reasons, patients were excluded if they were likely to suffer from severe or terminal illness, psychotic disorders, and dementia as inferred from their use of co-medication or at the discretion of the pharmacist e.g. in case of psychosocial problems. Eligible patients participated through filling out an online questionnaire. To reduce selection bias, face-to-face interviews using the TMI were conducted in the pharmacy with older people who were less likely to access the online questionnaire and with patients whose refill histories revealed that they had missed ≥ 1 prescriptions in the past year and who were therefore suspected to be non-adherent. For all patients, refill data were extracted from the automated dispensing records of the pharmacy. The medical ethical committee of the Leiden University Medical Center approved the study. All patients who participated online and in the pharmacy gave informed consent.

### Demographic and clinical characteristics

We assessed patients' demographic and clinical characteristics including duration of use. To distinguish between primary and secondary prevention, we assessed patients' indication for statin use which included elevated cholesterol levels and/or diabetes versus myocardial infarction, angina pectoris, transient ischemic attack, stroke and/or intermittent claudication or having undergone percutaneous coronary intervention and/or coronary bypass artery grafting.

### The Tailored Medicine Inventory

Development of the Tailored Medicine Inventory (TMI) has been described elsewhere. [3,4] Beliefs about the efficacy of statins were assessed with regard to prophylactic efficacy as well as experiences regarding knowledge and information about the efficacy (12 statements) [7–12] (see S1 Appendix). Responses to each of the statements were scored on five-point scales (0, fully disagree; 4, fully agree). Factor analysis (varimax rotation) and inspection of the item contents revealed 3 dimensions of which 2 had sufficient internal consistency, namely 'knowledge and information about the efficacy' and 'being convinced of the necessity' (see S1 Appendix).

Worry about side effects was assessed for known side effects of statins including: (a) muscular and joint pain or cramps, (b) vomiting or feeling nauseous, (d) intestinal complaints, (c) rash, (d) fatigue and (e) insomnia. [1, 13–14] Experiences with known side effects mentioned above and less-known side effects were assessed with a comprehensive checklist consisting of classes of side effects with regard to: (a) memory and concentration, [15–16] (b) emotional problems, [10, 15–18] (c) additional skin and hair problems, [18–20] (d) heart and veins, [15,17,20] (e) bladder, [18, 20] (f) gynecologic and sexual complaints (in women), [10–11,15, 17–18] (g) male sexual problems, [14, 20] (h) additional stomach problems, [15–17, 20] and (i) additional muscle complaints. [1]

**Fig 1:**
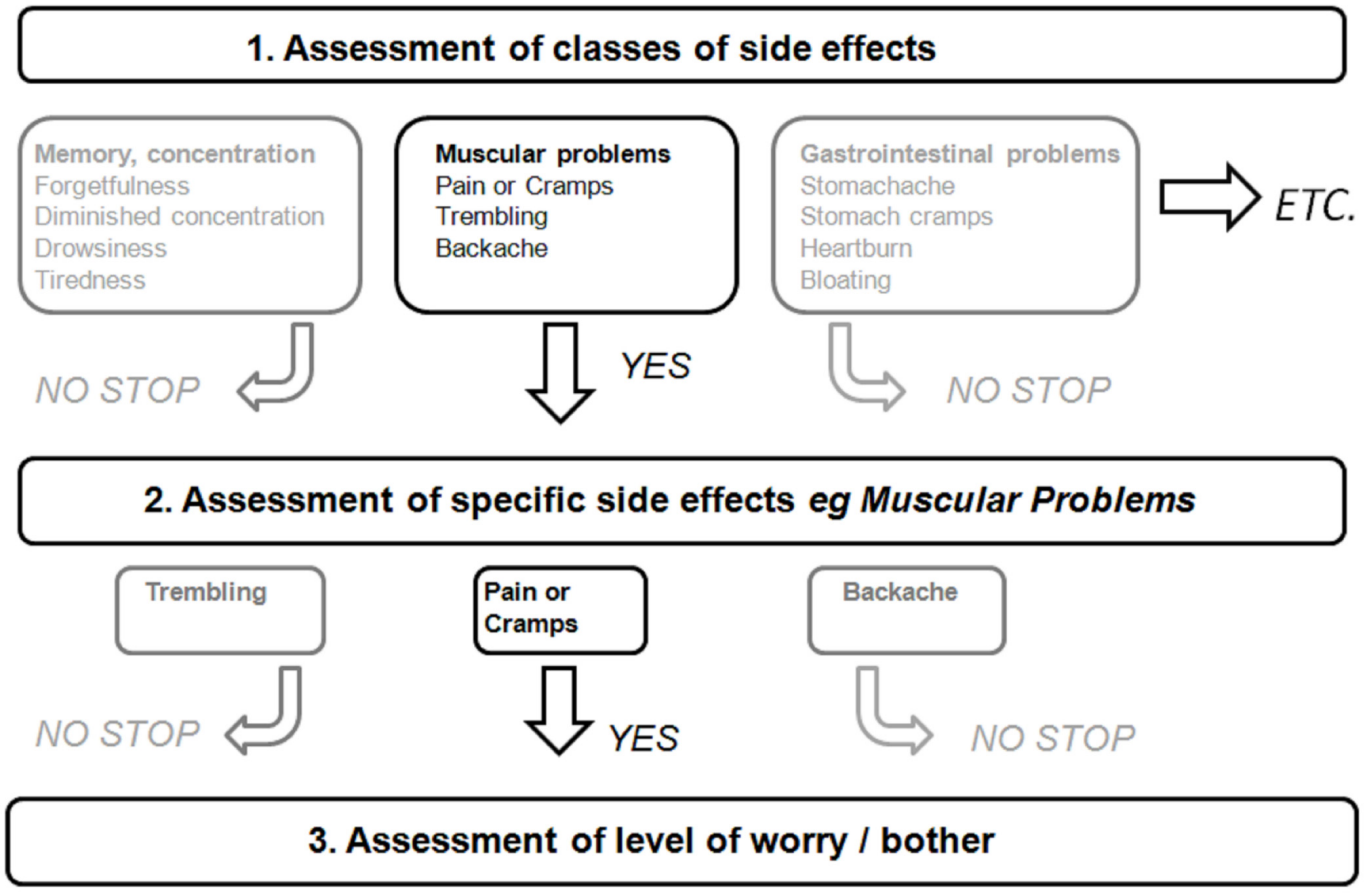
Example of tailored examination with the TMI.

To avoid test burden, experiences with regard to side effects were efficiently assessed in a three step manner enabled by logical routes (see Fig 1 for an illustration). First, patients had to indicate which class(es) of side effects they had experienced. To avoid reporting bias, the specific side effects belonging to a class were displayed in parentheses behind that class. Subsequently, for each endorsed class of side effects, patients had to indicate which specific side effect(s) they had experienced. Finally, only for the side effect(s) experienced, patients had to indicate the level of bother. Worries about side effects were also efficiently assessed but in a two step manner adopting steps 2 and 3 of the approach shown in Fig 1. Level of worrying and bother were assessed on five point scales (1, not bothersome/some worry; 5, very bothersome/very worried).

Practical problems of statin use were also assessed in the same tailored manner in which experiences with side effects were assessed. The comprehensive checklist of practical problems consisted of specific problems with regard to (a) (written) information, [6,17 21– 23] (b) logistical problems such as availability and getting refills, [11,24] (c) dosage and intake, [10,16,22,25] (d) packaging, [11,22, 24] and (e) other problems. [1] Here too, level of bother was assessed on a five point scale (1, not bothersome; 5, very bothersome).

### Perceived Self-efficacy

´Perceived self-efficacy´ with regard to 'learning about' and 'taking of' statins was assessed with the 8-item Medication Understanding and Use Self-efficacy (MUSE) scale. [6] Internal consistencies (alphas) were 0.85 for the previously reported dimension of ´perceived self-efficacy´ with regard to 'learning about' statins (4 items, scoring range 0–12) and 0.80 for 'taking of' statins (4 items, scoring range 2–12).

### Therapy Adherence

Non-adherence was assessed in the way as described elsewhere. [3] Self-reported non-adherence was assessed with the items from the Medication Adherence Rating Scale (MARS) [26] and the new Morisky Medication Adherence Scale (MMAS), [27] as well as additional items about forgetting to take statin tablets and persistence of taking statins. Factor analysis (varimax rotation), internal consistency measures, and inspection of the item contents supported the notion that the items measured 'unintentional non-adherence' due to forgetfulness and 'intentional non-adherence' or more deliberate non-adherence as underlying dimensions. Accordingly, for every patient, (un)intentional non-adherence total scores were calculated by summating the items measuring (un)intentional non-adherence (See S2 Appendix).

In addition, non-adherence was inferred from the pharmacy refill data. To that end, a Medication Possession Ratio (MPR) was calculated using information on statin dispensing data prior to the date on which patients were recruited for the study. We followed the methodology previously described by Gardarsdottir et al. [28] So-called 'treatment episodes' were calculated by allowing 90-day gaps between the theoretical end date of a statin prescription and a successive statin prescription. Where the dispensing date of the successive statin prescription preceded the theoretical end date of the prior prescription, this overlap was accounted for. For each patient, the MPR was calculated for the last treatment episode prior to recruitment.

### Statistical Analysis

Perceptions and experiences were summarized by means of descriptive statistics. The total scores of unintentional and intentional non-adherence were found to be skewed, and were therefore dichotomized at <80% versus ≥80% of the score distribution (unintentional non-adherence, score of 0 vs. ≥1; intentional non-adherence, score of ≤1 vs. ≥2). Likewise, the MPR was dichotomized (adherence ≥ 80% vs. non-adherence < 80%). Associations of non-adherence with patient perceptions and experiences were examined with logistic regression analysis. First, we conducted a series of univariate logistic regression analyses in which we included demographic and clinical characteristics, mode of participation (online vs. face-to-face interview), the extent of patients' knowledge about the efficacy and the extent to which they were convinced of the efficacy, the number of side effects patients were worried about or had experienced, the number of experienced practical problems as well as 'perceived self-efficacy'. Variables which had a univariate association with non-adherence (*P* < 0.10) were subsequently entered as independent variables into three multivariate logistic regression models with self-reported intentional and unintentional non-adherence, as well as the MPR calculated from the pharmacy refill data as the dependent variables. For each significant independent variable reflecting patient experiences and perceptions, we included an interaction term between that variable and 'perceived self-efficacy' with regard to 'learning about' and 'taking of' statins. All analyses were done with SPSS version 20 (See S3 Appendix for Dataset).

## Results

A total of 229 patients participated. Of them, 41 (18%) participated through a face-to-face interview. Table 1 presents the demographic and clinical characteristics of the patients.

**Table 1:** Demographic and clinical characteristics of the patients.

Variables	Statistic
N participants	229
**Demographic characteristics**	
n (%) men	146 (64)
mean age (SD) in years	63.9 (10.2)
n (%) married or living together	185 (81)
n (%) higher educated (vs. low to intermediate) *	105 (46)
**Clinical Characteristics**	
*Status of statin use*	
n (%) starters (< 3 months)	21 (9)
n (%) users (> 3 months)	189 (83)
n (%) discontinued	19 (8)
*Duration of use*	
*Users*	
n (%) 0–1 years	23 (12)
n (%) 1–4 years	64 (34)
n (%) 4 years or longer	102 (54)
*Discontinued*	
n (%) 0–1 years	6 (32)
n (%) 1–4 years	8 (42)
n (%) 4 years or longer	5 (26)
*Name of statin treatment*	
n (%) Atorvastatin	41 (18)
n (%) Fluvastatin	2 (1)
n (%) Pravastatin	19 (8)
n (%) Rosuvastatin	27 (12)
n (%) Simvastatin	140 (61)
*Primary vs*. *Secondary Prevention* **	
*Primary Prevention*	
n (%) elevated cholesterol	117 (51)
n (%) diabetes	37 (16)
*Secondary Prevention*	
n (%) MI/AP/ TIA/Stroke/ IC	81 (35)
n (%) PCI	31 (14)
n (%) CBAG	12 (5)

* originally scored on an 8-point scale (1, elementary education; 8, university) and dichotomized ≤ 4 (low to intermediate educational level) vs. ≥ 5 (higher educational level).

** total sum of reported percentages exceeds 100%, because patients could have >1 indication. Abbreviations: MI, Myocardial Infarction; AP, Angina Pectoris; TIA, Transient Ischemic Attack; IC, Intermittent Claudication, PCI, Percutaneous Coronary Intervention; CBAG, Coronary Bypass Artery Grafting.

Contrasting patients who (fully) disagreed with or were neutral (score ≤ 2 on 5-point scale) versus those patients who (fully) agreed (score ≥3 on 5-point scale) with various statements about the efficacy showed that substantial numbers of patients did not believe the use of statins to be necessary (N = 92, 40%), were not convinced of (N = 109, 48%) or doubted the efficacy (N = 108, 47%), did not believe statins to prevent (recurrence of) cardiovascular disease (N = 112, 49%) or thought the efficacy to be limited (N = 163, 71%). Although 194 (85%) patients reported that they knew why they needed to use statins, 131 (57%) patients did not know how statins worked, 116 (51%) patients lacked information thereof, and 101 (44%) patients did not know to what extent statins reduced the risk of heart disease, but 99 (43%) also disagreed with the statement that "You have to believe that statins work, otherwise you might just as well not use them".

Table 2 presents the side effects associated with statins (both worry and experience assessed) and other side effects (only experience assessed). Over a quarter to a third of patients expressed worry about ache, stiffness, swelling or inflammation of joints, and muscular pain or muscle cramps. Fewer patients worried about intestinal complaints, fatigue and insomnia and few patients worried about vomiting or feeling nauseous and rash (see Table 2). The mean level of worry was on average moderate to high. No clear differences in mean levels of evoked worry were observed for the various side effects. At the same time, individual differences in evoked worry were observed. A fifth of the patients had experienced well-known side effects of statins including ache, stiffness, swelling or inflammation of joints and muscular pain or muscle cramps (see Table 2). Some less-known side effects of statins that were frequently experienced included decreased erection (in males), a dry mouth, and backache. The mean level of bother was on average moderate to high.

**Table 2:** Worry about and experience of side effects associated with statin use.

	Side Effects			
	Worry		Experience	
	Yes n (%)	M level (SD)	Yes n (%)	M level (SD)
**Side effects associated with statins**				
Ache, stiffness, swelling joints	62 (27)	3.6 (1.4)	46 (20)	4.6 (0.6)
Muscular pain or muscle cramps	80 (35)	3.7 (1.4)	56 (25)	4.6 (0.8)
Vomiting or feeling nauseous	6 (3)	3.7 (0.8)	3 (1)	3.7 (1.2)
Intestine complaints	35 (15)	3.3 (1.4)	20 (9)	4.8 (0.4)
Rash	19 (8)	3.1 (1.2)	14 (6)	4.1 (1.0)
Fatigue	34 (15)	2.9 (1.2)	18 (8)	4.5 (0.7)
Insomnia	33 (14)	3.2 (1.4)	26 (11)	4.4 (0.9)
**Other side effects**				
Memory, concentration or tiredness				
Sleepiness or drowsiness			7 (3)	4.7 (0.8)
Forgetfulness			14 (6)	4.5 (0.8)
Diminished concentration			15 (7)	4.7 (0.6)
Gynecological complaints				
Painful or sensitive breasts			3 (1)	4.0 (1.0)
Vaginal discharge, dryness or itch			3 (1)	3.7 (1.5)
Hot flashes			6 (3)	3.7 (1.2)
Decreased libido			8 (4)	3.5 (1.2)
Male sexual problems				
Decreased ejaculation			16 (7)	3.4 (1.5)
Decreased erection			27 (12)	4.1 (1.3)
Decrease libido			13 (6)	3.9 (1.3)
Mood				
Feeling restless			11 (5)	4.2 (0.9)
Emotional bluntness			5 (2)	4.0 (0.7)
Gloomy mood			9 (4)	4.1 (1.0)
Anxiety, panic or insecurity			7 (3)	4.7 (0.8)
Skin or hair				
Hair loss			8 (4)	3.5 (1.1)
Sweating			16 (7)	4.1 (1.0)
Heart, vessels or bladder				
Heart palpitations			6 (3)	4.7 (0.8)
Orthostatic hypotension			10 (4)	3.7 (1.3)
Edema			14 (6)	3.8 (1.4)
Urinary retention			4 (2)	3.8 (1.0)
Incontinence			13 (6)	4.2 (1.2)
Mouth, stomach or intestines				
Ache, cramps, heartburn or bloating			16 (7)	4.6 (0.9)
Dry mouth			31 (14)	3.8 (1.2)
Muscles, bones or joints				
Trembling			3 (1)	3.0 (1.7)
Backache			28 (12)	4.5 (0.6)

Eighty-seven patients (38%) had experienced one or more practical problems. The most frequently experienced practical problems were those with regard to information, intake of tablets, and package (N = 53, 23%) and limitations in daily life (N = 35, 15%). Logistical problems such as getting insufficient statin tablets (N = 17, 7%) and concerns about medication interactions were less often reported (N = 27, 12%). On average, practical problems posed moderate hindrance to patients (mean level of hindrance varying from 2.5–3.5). Again, variation in level of hindrance between patients was observed (standard deviations of hindrance levels varying from 1.1–1.3). The mean score of 'perceived self-efficacy' with regard to 'learning about' statins was 10.0 (standard deviation 2.5) and with regard to 'taking of' statins it was 10.9 (standard deviation 1.9).

Turning to non-adherence, the likelihood of *un*intentional non-adherence was increased by 1.5 fold for each experienced practical problem. The likelihood of *in*tentional non-adherence was increased by 1.9 fold for each additional side effect worried about. Higher 'perceived self-efficacy' with regard to ' taking statins' was associated with both a decreased likelihood of unintentional and intentional non-adherence (See Table 3). However, 'perceived self-efficacy' did not weaken the aforementioned associations between non-adherence and negative perceptions and experiences (results not shown). No associations were found between patient perceptions and experiences with the MPR calculated from the pharmacy refill data.

**Table 3:** Multivariate Logistic Regression: Prediction of self-reported unintentional and intentional non-adherence and non-adherence measured with the Medication Possession Ratio by patients' perceptions and experiences.

	Self-reported Non-adherence		Refill Non-adherence
Predictors	Unintentional ^a^	Intentional ^b^	MPR ^c^
	Odds ratio *(95%CI)*	Odds ratio *(95%CI)*	Odds ratio *(95%CI)*
Age	0.98 (0.94–1.02)		
Duration of use	0.58 (0.34–0.98)*		1.91 (0.92–3.94)
Primary vs. secondary prevention			
Primary prevention		*Reference Category*	
Secondary prevention		0.44 (0.17–1.10)	
*Efficacy*:			
Being convinced of necessity		0.93 (0.80–1.08)	0.88 (0.74–1.04)
*Side effects*:			
Number of side effects worried about		1.90 (1.17–3.08) †	
Number of side effects experienced		0.94 (0.71–1.23)	
*Practical problems*:			
Number of practical problems	1.54 (1.13–2.10) †		
Learning 'perceived self-efficacy' (MUSE)	1.06 (0.87–1.30)		
Taking 'perceived self-efficacy' (MUSE)	0.55 (0.42–0.73) †	0.76 (0.62–0.92) †	0.85 (0.69–1.05)

Patients included in the analysis:

^a & b^: N = 192;

^c^: N = 190

* P < 0.05;

^†^ P < 0.01; MPR, Medication Possession Ratio; CI, Confidence Interval; MUSE, Medication Understanding and Use Self-efficacy Scale.

## Discussion

Overall, our findings showed that a substantial number of patients faced a wide array of specific barriers to appropriate statin use. Many patients doubted the necessity of statins and lacked information about and knowledge of the efficacy of statins. There was often worry about and experience of side effects, in particular the known muscle and joint side effects but also less-known side effects such as decreased erection (in males), a dry mouth, and backache. Worry about side effects was found to be associated with increased intentional non-adherence. Practical problems were also experienced and were associated with increased unintentional non-adherence.

Contrary to our prediction, 'perceived self-efficacy' did not moderate, or specifically weaken, the associations between non-adherence and negative perceptions and experiences regarding the efficacy, the side effects, and the practical problems. This finding is consistent with our previous findings in patients treated with antidepressants [3] and women treated with endocrine therapy for prevention of breast cancer recurrence. [4]

Generally, our findings were consistent with previous findings yet also expand these in several regards and point to fruitful avenues for further research as well. First, our study of specific barriers can be considered to be complementary to previous studies conducted in various patient groups which adopted a patient oriented perspective but which addressed general beliefs and characteristics. [5,9,10] Second, given that we found a positive association between worry about side effects and intentional non-adherence and that less-known side effects of statins were also reported occasionally, assessments of side effects which are typically aimed at actual experience of known side effects of statins [1, 12, 13] should be expanded with both worry about side effects and less obvious side effects. Third, practical problems deserve also further attention as these were associated with unintentional non-adherence. Although several studies addressed practical problems, these were limited to specific ones. [1,23,24] A more comprehensive list of practical problems, such as the one we examined, is therefore recommended.

Our findings underline the potential of the TMI as an impetus to improving the communication about appropriate use of statins and adherence between clinicians and patients. Because the TMI assesses a wide range of potential problems from the patient perspective, it is likely to prevent patronizing of patients. [16] Furthermore, as the TMI is web-based, it can be easily filled out by a patient prior to the consultation with a physician or pharmacist. Alternatively, it could be adopted for use in the waiting room. Further implementation research must be conducted to reveal how much shortening and simplification of the TMI is needed and how the TMI should be applied in clinical practice. A next logical step would be to let clinicians and patients formulate practical solutions to problems revealed by the TMI. The TMI can aid clinicians in tailoring adherence improving interventions to the needs of individual patients. Some patients might need support in coping with worry about or experience of side effects or practical problems. Other patients could benefit from additional education about the efficacy of statins. [29] Indeed, a more subtle problem unraveled by the TMI was the finding on the one hand that the majority of patients indicated that they knew why they needed to use statins, while on the other hand substantially less people knew how statins worked, lacked information thereof, or did not know to what extent statins reduced the risk of heart disease. [30] In future, such individually tailored interventions might reduce the substantial non-adherence to statins [1].

This study had several strengths. A key strength was the use of the TMI. Because the TMI skips irrelevant items, it enables assessing a wide array of perceptions and experiences in a manner that is both comprehensive and efficient. The TMI thereby reduces the chance of overlooking crucial perceptions and experiences. Because the causal structure of non-adherence seems to be multifaceted [2] in the sense that different negative perceptions and/or experiences may explain non-adherence for different patients, assessing the wide array of perceptions and experiences seems unavoidable. At the same time, the TMI is likely to prevent question burden in patients who do not have experienced many side effects or practical problems. A specific strength with regard to the assessment of side effects was the use of a comprehensive checklist. We believe that for the understanding of adherence from the perspective of patients, it is not relevant to distinguish between 'true' side effects that can be traced back to the pharmacodynamic mechanisms through which statins exert their effects and 'false' side effects reflecting unrelated symptoms. After all, adherence behavior is likely to be governed by what patients believe, regardless of whether this is 'true' or 'false'. Moreover, unlikely side effects could also reflect unknown side effects or unexpected reactions of the body to statins. Finally, in line with previous recommendations, [2] a strength with regard to the assessment of adherence was that we adopted both self-reported adherence and adherence as inferred from pharmacy refill data. While self-reported adherence is believed to be liable to social desirability bias, it allows to distinguish between non-adherence of a more unintentional and intentional nature. Although pharmacy refill data are likely to be a more objective measurement of adherence, such a distinction is not rendered easily thereby making adherence inferred from pharmacy refill data a more crude overall adherence measure that reflects both unintentional and intentional non-adherence. The fact that differential associations were observed between patient perceptions and experiences with self-reported unintentional and intentional non-adherence, but no associations were found with adherence inferred from pharmacy refill data, are also interesting in this regard. They actually contradict the liability of self-reported non-adherence to social desirability bias and at the same time are actually in favor of the idea that adherence measured from pharmacy refill data constitute a crude measurement. Clearly more research about this matter is warranted.

Several limitations of this study deserve to be mentioned as well. First, we relied on self-report to assess clinical characteristics and the findings could be liable to recall bias. However, as discussed in the previous paragraph, this may be less of a problem for self-reported adherence than mainstream paradigms suggest. Second, although we could infer non-adherence from refill data, we had no medical data of patients. Therefore, we could not assess to what extent non-adherence reflected non-acceptance on legitimate grounds e.g. a very low prophylactic efficacy for a particular patient coupled with severe muscle side effects and other impairments in everyday life. Third, our cross-sectional study design precluded an examination of incident non-adherence and non-persistence and changes in perceptions and experiences over time. Examination of a wide array of perceptions and experiences in longitudinal studies that also enroll a greater number of patients who initiate statin therapy is therefore recommended. Fourth, because of patient anonymity issues and because patients were only informed by mail about the study, we decided not to record non-response. Although response bias may have played a role, we deemed it not to be a key concern because our objective was not to estimate the prevalence of patients' perceptions and experiences regarding the efficacy, side effects and practical problems and we also observed ample variance in demographic and clinical characteristics.

Taken together, we conclude that insight with the TMI into patients' specific barriers with regard to appropriate statin use, is likely to reveal personal reasons for non-adherence that may be dealt with by the clinician-patient alliance to overcome non-adherence.

## Supporting Information

S1 Appendix Items to assess experiences and perceptions with regard to the efficacy of statins. (DOC)

S2 Appendix Items to assess unintentional and intentional non-adherence. (DOC)

S3 Appendix Dataset. (SAV)
