# Supplementary material for: Understanding Statin Non-Adherence: Knowing Which Perceptions and Experiences Matter to Different Patients
Source: PLoS One. 2016 Jan 25;11(1):e0146272. doi: 10.1371/journal.pone.0146272 (PMC4726652; doi:10.1371/journal.pone.0146272)
Supplement: S1 Appendix — (DOC) [file pone.0146272.s001.doc]

| **S1 APPENDIX** Items to assess experiences and perceptions with regard to the efficacy of statins | | | | | |
| --- | --- | --- | --- | --- | --- |
|  | Factors | | | | |
| Items & their factor loadings | I^a^ |  | II^b^ |  | III^c^ |
| 1. Statins prevent me from getting heart disease in future |  |  | 0.79 |  |  |
| 2. The use of statins is necessary for me |  |  |  |  | 0.57 |
| 3. I am convinced of the efficacy of statins |  |  |  |  | 0.59 |
| 4. I have doubts about the efficacy of statins R |  |  |  |  | 0.74 |
| 5. I know how statins work | 0.84 |  |  |  |  |
| 6. I received information about how statins work | 0.78 |  |  |  |  |
| 7. I know why I have to use statins ** | 0.56 |  |  |  |  |
| 8. Statins have a limited efficacy R * |  |  |  |  |  |
| 9. You have to believe that statins work, otherwise you might just as well not use them R ** |  |  |  |  | 0.68 |
| 10. I know to what extent statins reduce the chance of heart disease | 0.75 |  |  |  |  |
| 11. By using statins, I do everything to prevent heart disease (again) |  |  | 0.78 |  |  |
| 12. The longer you use statins, the better they work * |  |  |  |  |  |
| % of variance explained | 32 |  | 13 |  | 10 |
| Cronbach's alpha | 0.78 |  | 0.68 |  | 0.73 |
| Interpretation of factor dimensions: a = Knowledge and information about the efficacy, b = Prevention, c = Being convinced of the necessity. Notes: * factor could not be interpreted ** item deleted to improve internal consistency (alpha) of dimension. | | | | | |
