# Supplementary material for: Understanding Statin Non-Adherence: Knowing Which Perceptions and Experiences Matter to Different Patients
Source: PLoS One. 2016 Jan 25;11(1):e0146272. doi: 10.1371/journal.pone.0146272 (PMC4726652; doi:10.1371/journal.pone.0146272)
Supplement: S2 Appendix — (DOC) [file pone.0146272.s002.doc]

| **S2 Appendix** Items to assess unintentional and intentional non-adherence | | | | |
| --- | --- | --- | --- | --- |
|  |  | Factors | | |
| Factor loadings of adherence items |  | Intentional non-adherence |  | Unintentional non-adherence |
| *MMAS items* |  |  |  |  |
| 2. Over the past 2 weeks, were there any days when you did not take your statins? § |  | 0.36 |  | 0.31 |
| 3. Have you ever cut back or stopped taking your statins without telling your doctor because you felt worse when you took it? |  | 0.48 |  | 0.04 |
| 4. When you travel or leave home, do you sometimes forget to bring along your statins? § |  | 0.23 |  | 0.40 |
| 5. Did you take your statins yesterday R? ‡ |  | -0.08 |  | 0.15 |
| 7. Taking medication everyday is a real inconvenience for some people. Do you ever feel hassled about sticking to your statin treatment plan? § |  | 0.24 |  | 0.51 |
| 8. How often do you have difficulty remembering to take all your statins? || |  | 0.09 |  | 0.87 |
| *MARS items ||* |  |  |  |  |
| 1*.* I forget to use my statins |  | 0.10 |  | 0.67 |
| 2. I adjust the dosage of my statins |  | 0.78 |  | -0.05 |
| 3. I stop using statins for a while |  | 0.68 |  | 0.17 |
| 4. I decide to skip a statin dose |  | 0.76 |  | 0.11 |
| 5. I take less statin tablets than prescribed to me |  | 0.74 |  | -0.01 |
| *Additional* items |  |  |  |  |
| 1. I sometimes forgot to take my statins |  | 0.12 |  | 0.75 |
| 2. I forgot whether I had already taken my statins § |  | 0.09 |  | 0.53 |
| 3. I intend to use statins in the way as prescribed ‡ |  | 0.29 |  | 0.11 |
| 4. I intend to prematurely discontinue using statins R ‡ |  | 0.30 |  | 0.13 |
|  |  |  |  |  |
| % of variance explained |  | 24 |  | 13 |
| Cronbach's alpha |  | 0.75 |  | 0.77 |
|  |  |  |  |  |
| *Excluded items:* |  |  |  |  |
| *Do you sometimes forget to take your statins? *(MMAS item 1)* |  |  |  |  |
| *When you feel like your symptoms are under control, do you sometimes stop taking your statins?(MMAS item 6)* *†* |  |  |  |  |
| *Notes: * MMAS item excluded from analysis because it was considered to be a duplicate item, † not applicable to statins, ‡ excluded because of low factor loading, § item deletion improves internal consistency (alpha) of dimension, || item(s) dichotomized; Items were excluded from the total score if they were duplicate, were not applicable, had low or ambiguous factor loadings, or when their deletion improved the internal consistency (alpha) of the dimension. The final calculation of the total scores was as follows:*  ***intentional non-adherence*** *= MMAS i3 + MARSi2 + MARSi3 + MARSi4 + MARSi5*  ***unintentional non-adherence*** *=**MMASi8 + MARSi1 + Additional i1* | | | | |
